# Change in Neutrophil-to-lymphocyte ratio (NLR) in response to immune checkpoint blockade for metastatic renal cell carcinoma

**DOI:** 10.1186/s40425-018-0315-0

**Published:** 2018-01-22

**Authors:** Aly-Khan A. Lalani, Wanling Xie, Dylan J. Martini, John A. Steinharter, Craig K. Norton, Katherine M. Krajewski, Audrey Duquette, Dominick Bossé, Joaquim Bellmunt, Eliezer M. Van Allen, Bradley A. McGregor, Chad J. Creighton, Lauren C. Harshman, Toni K. Choueiri

**Affiliations:** 1 Lank Center for Genitourinary Oncology, Dana-Farber Cancer Institute, 450 Brookline Avenue, Dana 1230, Boston, MA 02215 USA; 2 Department of Biostatistics and Computational Biology, Dana-Farber Cancer Institute, 450 Brookline Avenue, Boston, MA 02215 USA; 3 Emory University School of Medicine, 100 Woodruff Circle, Atlanta, GA 30322 USA; 4 Department of Imaging, Dana-Farber Cancer Institute & Department of Radiology, Brigham and Women’s Hospital, 450 Brookline Avenue, Boston, MA 02215 USA; 5 The Eli and Edythe L. Broad Institute of MIT and Harvard, 415 Main St, Cambridge, MA 02142 USA; 6 Department of Medicine, Dan L. Duncan Comprehensive Cancer Center, and Human Genome Sequencing Center, Baylor College of Medicine, One Baylor Plaza MS 305, Houston, TX 77030 USA

**Keywords:** Neutrophil-to-lymphocyte ratio, PD-1/pd-L1 PD-L1, Immunotherapy, Renal cell carcinoma, Prognostic biomarker

## Abstract

**Background:**

An elevated Neutrophil-to-lymphocyte ratio (NLR) is associated with worse outcomes in several malignancies. However, its role with contemporary immune checkpoint blockade (ICB) is unknown. We investigated the utility of NLR in metastatic renal cell carcinoma (mRCC) patients treated with PD-1/PD-L1 ICB.

**Methods:**

We examined NLR at baseline and 6 (±2) weeks later in 142 patients treated between 2009 and 2017 at Dana-Farber Cancer Institute (Boston, USA). Landmark analysis at 6 weeks was conducted to explore the prognostic value of relative NLR change on overall survival (OS), progression-free survival (PFS), and objective response rate (ORR). Cox and logistic regression models allowed for adjustment of line of therapy, number of IMDC risk factors, histology and baseline NLR.

**Results:**

Median follow up was 16.6 months (range: 0.7–67.8). Median duration on therapy was 5.1 months (<1–61.4). IMDC risk groups were: 18% favorable, 60% intermediate, 23% poor-risk. Forty-four percent were on first-line ICB and 56% on 2nd line or more. Median NLR was 3.9 (1.3–42.4) at baseline and 4.1 (1.1–96.4) at week 6. Patients with a higher baseline NLR showed a trend toward lower ORR, shorter PFS, and shorter OS. Higher NLR at 6 weeks was a significantly stronger predictor of all three outcomes than baseline NLR. Relative NLR change by ≥25% from baseline to 6 weeks after ICB therapy was associated with reduced ORR and an independent prognostic factor for PFS (*p* < 0.001) and OS (*p* = 0.004), whereas a decrease in NLR by ≥25% was associated with improved outcomes.

**Conclusions:**

Early decline and NLR at 6 weeks are associated with significantly improved outcomes in mRCC patients treated with ICB. The prognostic value of the readily-available NLR warrants larger, prospective validation.

## Background

The treatment paradigm for metastatic renal cell carcinoma (mRCC) has evolved over the last two decades from an era of cytokine regimens to VEGF targeted therapies (VEGF-TT) [1]. Prognostic models have also been refined over time, from the Memorial Sloan-Kettering Cancer Center criteria, which was initially established based on mRCC patients treated with interferon alfa (IFN-α) on clinical trials [2, 3], to the International Metastatic Renal Cell Carcinoma Database Consortium (IMDC) criteria, which has been validated in the era of oral VEGF-TT [4]. More recently, the treatment landscape has expanded to include contemporary immune checkpoint blockade (ICB). Nivolumab, a programmed cell death protein-1 (PD-1) antibody, became the first checkpoint inhibitor to be approved by the US Food and Drug Administration for second line mRCC in November 2015 [5, 6] and was subsequently approved for use in Europe [7]. Currently, there are several Phase 3 randomized trials investigating PD-1/PD-L1-based therapies in the first-line setting (NCT02420821, NCT02684006, NCT02811861). The first to result – the CheckMate-214 study – was stopped early due to a survival benefit seen with combination ICB versus single agent VEGF-TT [8]. Well-validated biomarkers that can be earlier prognostic or predictive readouts in mRCC treated with conventional ICB are lacking and represents an unmet need.

Inflammation is a recognized hallmark of cancer, however, the mechanism by which inflammation leads to worse outcomes in mRCC is not well described [9]. Neutrophilia is thought to occur as an inflammatory response and may lead to suppression of cytolytic activity of immune cells such as lymphocytes, natural killer cells, and activated T cells [10, 11]. An elevated neutrophil-to-lymphocyte ratio (NLR) has been shown to be associated with a poor prognosis in several solid tumors [12–14]. For example, in mRCC patients treated with VEGF-TT, an elevated NLR (NLR > 3) at baseline and an increase in NLR at 6-weeks were associated with worse outcomes in terms of survival and objective response rates [15]. The prognostic value of NLR in the current era of ICB has been evaluated in small subsets of patients, for example, with lung, melanoma, and bladder malignancies [16–18]. However, the utility of NLR in the context of contemporary immunotherapy for mRCC has not been well-defined. In this study, we investigated the association of baseline NLR, and changes during treatment, with outcomes in mRCC patients treated with conventional ICB.

## Methods

### Patients and data collection

We performed a retrospective analysis of all patients treated with anti-PD-1/PD-L1-based treatment regimens at the Dana-Farber Cancer Institute (DFCI) from 2009 to 2017. For patients who have received multiple ICB-based regimens, clinical information was only included from the earlier systemic treatment line. Data collected from patient’s electronic medical records included demographic information, smoking status, histology (including percent sarcomatoid and rhabdoid differentiation), International Metastatic Renal Cell Carcinoma Database Consortium (IMDC) risk factors (hemoglobin < lower limit of normal, corrected calcium > upper limit of normal (ULN), platelets > ULN, absolute neutrophil count < ULN, Karnofsky Performance Status (KPS) < 80%, and time from diagnosis to systemic treatment <1 year), lactate dehydrogenase level, Neutrophil-to-Lymphocyte ratio (NLR) at start of IO and at 6 (±2) weeks after therapy initiation, drug target (PD-1, PD-L1), line of systemic therapy, duration of treatment, best response to PD-1/PD-L1, scan date of progression, date of death or last follow up. Response and progression was determined per Response Evaluation Criteria in Solid Tumor version 1.1 [19] by centralized review for patients treated on clinical trials or by an expert radiologist (KMK).

### Statistical analysis

Categorical variables, such as patient and disease characteristics, were described using frequencies and percentages. Quantitative variables were presented as medians and ranges. Kruskal-Wallis test was used to compare baseline NLR levels by patient and disease groups. We first investigated the impact of baseline NLR (as natural log-transformed [lnNLR]) on objective response rate (ORR: complete response or partial response), progression free survival (PFS), and overall survival (OS) using logistic or Cox regression models, adjusted for line of therapy, number of IMDC risk factors, and histology (clear cell RCC vs. non-clear cell RCC). PFS was defined as the time from the first dose of anti-PD-1/PD-L1 to radiographic or clinical progression or death, whichever came first, censored at last follow-up for patients who have not progressed. OS was calculated as the time from the first dose of PD-1/PD-L1 inhibitor to the date of death or last follow-up. Martingale residuals plots were used to verify the linear assumption of the Cox models for the continuous LnNLR values.

A landmark analysis at 6 weeks was conducted to explore the prognostic value of LnNLR at 6 (±2) weeks or relative NLR change (calculated as % change ({[NLR week 6 / NLR week 0] - 1}*100) and subsequently grouped in three groups (≥25% decrease, no change [<25% decrease to <25% increase], ≥25% increase) on OS, PFS, and ORR. For the landmark analysis, PFS and OS were calculated from 6 weeks after anti-PD-1/PD-L1 initiation.

SAS version v9.4 (Cary, NC, USA) was used to carry out the above analysis. All statistical tests were two-sided and statistical significance was considered at *p* < 0.05.

## Results

### Baseline characteristics and outcomes

This analysis included 142 patients who received anti-PD-1/PD-L1-based treatments at DFCI. Baseline patient and disease characters are provided in Table 1. The median age was 61 years (range: 22–82), 84.5% (*n* = 120) of patients had clear cell histology, and 15.5% (*n* = 22) had sarcomatoid differentiation. Approximately 60% (*n* = 85) of the patients were intermediate risk, while 18.3% (*n* = 26) were favorable risk and 21.8% (*n* = 31) were poor risk. Sixty-two patients (43.7%) received treatment in the first-line setting, 37 (26.1%) in the second-line, and 43 (30.3%) received treatment in the third-line or later, with the majority receiving ICB monotherapy (*n* = 76, 53.5%). Overall, 91 patients received PD-1 based therapy (71 monotherapy, 20 combination therapy) and 51 received PD-L1 based therapy (5 monotherapy, 46 combination therapy). Forty-six patients received standard of care PD-1 monotherapy and 96 were treated as part of a clinical trial.

**Table 1:** Patient and disease characteristics (*N* = 142)

	Number	Percent
Age at start of anti-PD-1/PD-L1 therapy		
< 60	58	40.8
≥ 60	84	59.2
Smoker		
No	69	48.6
Yes	72	50.7
Unknown	1	0.7
Gender		
Female	41	28.9
Male	101	71.1
Histology		
Clear cell RCC	120	84.5
Non-clear cell RCC	22	15.5
Presence of Sarcomatoid		
No	119	83.8
Yes	22	15.5
Unknown	1	0.7
Presence of Rhabdoid		
No	134	94.4
Yes	6	4.2
Unknown	2	1.4
IMDC risk group at start of anti-PD-1/PD-L1 therapy		
Favorable	26	18.3
Intermediate	85	59.9
Poor	31	21.8
Line of therapy		
1	62	43.7
2	37	26.1
≥ 3	43	30.3
Type of anti-PD-1/PD-L1 therapy		
Monotherapy	76	53.5
Combination therapy PD-L1 + VEGF targeted therapy (*n* = 46) PD-1 + VEGF-targeted therapy (*n* = 9) PD-1 + CTLA-4 (*n* = 7) PD-1 + other (n = 4)	66	46.5
	Median	range
NLR - baseline	3.9	1.3–42.4
NLR - week 6	4.1	1.1–96.4

The median NLR at baseline was 3.9 (range: 1.3–42.4) and 4.1 (range: 1.1–96.4) at 6 (±2) weeks. Median follow-up since initiation of the anti-PD-1/PD-L1 treatment was 16.6 (range: 0.7–67.8) months. Median duration on therapy was 5.1 (range: <1–61.4) months and 106 (74.6%) patients had discontinued the anti-PD-1/PD-L1-based treatments at time of analysis. The observed ORR rate was 31% (*N* = 44, 95% confidence interval (CI) 24–39). Median PFS and OS after therapy initiation were 7.3 (95% CI 3.5–8.8) months and 29.6 (95% CI 21.1–59.2) months, respectively.

### Associations of NLR with baseline characteristics

**Fig. 1:**
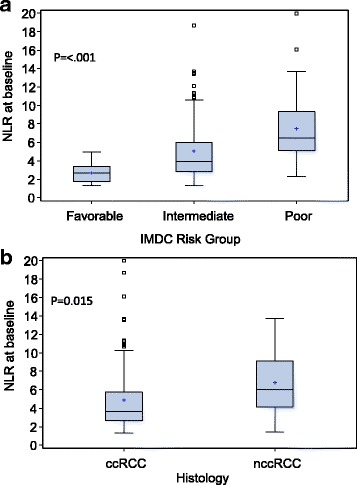
Neutrophil-to-lymphocyte ratio (NLR) at start of anti-PD-1/PD-L1 therapy by **a** IMDC risk groups and **b** Histology (clear cell RCC, ccRCC; non-clear cell RCC, nccRCC)

Baseline NLR levels were significantly higher in the poor IMDC risk group compared to those with favorable or intermediate risk (*p* < 0.001, Fig. 1a). Similarly, patients with non-clear cell histology had elevated NLR compared to those with clear cell histology (*p* = 0.015, Fig. 1b). We did not detect significant association of baseline NLR with other patient characteristics such as age, gender, smoker status, and line of therapy (*p*-values >0.15, data not shown).

### Prognostic role of pre-treatment NLR

The association of baseline NLR and treatment outcomes is presented in Table 2. Martingale residual plots confirmed the linearity of LnNLR (data not shown). A higher baseline NLR showed a trend toward lower ORR (adjusted odds ratio (OR) per 1 unit increase in lnNLR = 0.49, 95% CI 0.22–1.09, *p* = 0.081), shorter PFS (adjusted hazard ratio (HR) per 1 unit increase in lnNLR = 1.80, 95% CI 1.14–2.86, *p* = 0.012), and shorter OS (adjusted HR per 1 unit increase in lnNLR = 1.70, 95% CI 0.99–2.94, *p* = 0.056). The association of NLR at baseline and outcomes were consistent by type of ICB therapy (PD-1 vs PD-L1 based treatment) and by line of therapy (first line vs second line and beyond, data not shown).

**Table 2:** Association of NLR at baseline, at 6-weeks, and change at week 6 (±2 weeks) with treatment outcomes in multivariable Cox and Logistic regression models

	ORR (CR + PR)			PFS			OS		
	Total N/ N response	Adjusted-OR^b^	*p*-value	Total N/ N event	Adjusted-HR^b^	*p*-value	Total N/ N event	Adjusted-HR^b^	*p*-value
Continuous Ln(NLR) [baseline]	142/44	0.49 (0.22–1.09)	0.081	142/96	1.80 (1.14–2.86)	**0.012**	142/51	1.70 (0.99–2.94)	0.056
Continuous Ln(NLR) [6-weeks]^a^	134/44	0.22 (0.10–0.52)	**0.001**	117/72	3.61 (2.21–5.88)	**<0.001**	134/46	2.51 (1.71–3.69)	**<0.001**
NLR-change [6-weeks]^a^									
Decrease ≥25%	28/12	1.52 (0.49–4.68)	0.112	27/13	0.55 (0.26–1.18)	**<0.001**	28/6	0.33 (0.12–0.88)	**0.004**
No change	58/21	1.00 (reference)		53/30	1.00 (reference)		58/18	1.00 (reference)	
Increase ≥25%	48/11	0.45 (0.18–1.16)		37/29	2.60 (1.53–4.39)		48/22	1.57 (0.83–2.99)	

*Abbreviations*: *ORR* objective response rate, *CR* complete response, *PR* partial response, *OR* odds ratio, *PFS* progression free survival, *HR* hazard ratio, *OS* overall survival

^a^Landmark approach was used where OS and PFS were calculated from 6 weeks after therapy initiation. Patients who progressed before the 6 week landmark time were excluded for PFS analysis

^b^Adjusted for line of therapy, number of IMDC risk factors, histology (clear cell RCC vs non-clear cell RCC); baseline Ln(NLR) was also included as a covariate for the analysis of NLR change at 6 weeks. Boldface numerical values indicate statistically significant results

### Prognostic role of NLR at 6 (±2) weeks

**Fig. 2:**
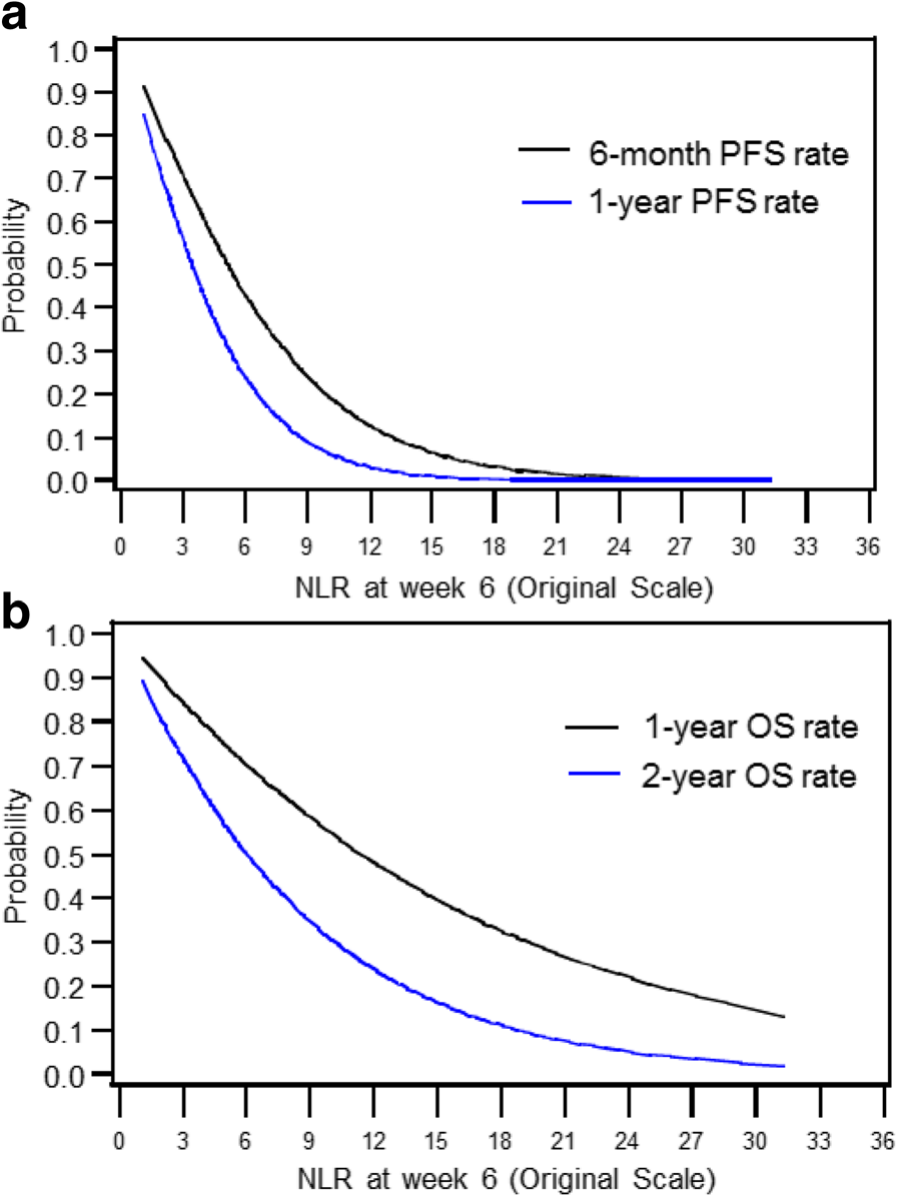
Estimated **a** 6-month and 1-year PFS rate, and **b** 1- and 2-year OS rate from Cox regression based on continuous neutrophil-to-lymphocyte ratio (NLR) at week 6 (±2 weeks). NLR was modeled on the natural logarithmic scale and transformed back to the original scale for graphic presentation. PFS and OS were calculated from 6 weeks of therapy

**Fig. 3:**
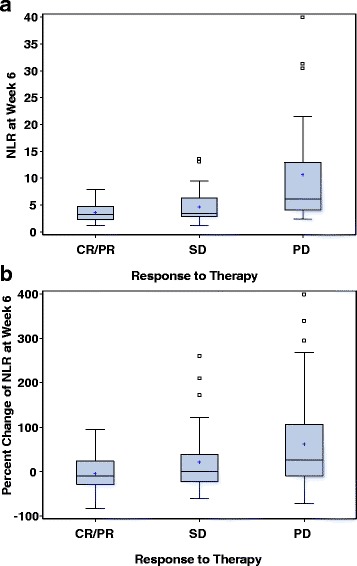
Neutrophil-to-lymphocyte ratio (NLR) at week 6 (**a**) and percent (%) change of NLR at week 6 (**b**) according to response to therapy (complete/partial response, CR/PR; stable disease, SD; progressive disease, PD)

NLR at 6 weeks was a stronger predictor of all three outcomes (ORR, PFS, and OS) than baseline NLR (Table 2, Fig. 2). A higher 6 (±2) week NLR was independently associated with a lower ORR (Fig. 3a; adjusted OR per 1 unit increase in lnNLR = 0.22, 95% CI 0.10–0.52, *p* = 0.001), shorter PFS (adjusted HR per 1 unit increase in lnNLR = 3.61, 95% CI 2.21–5.88, *p* < 0.001), and shorter OS (adjusted HR per 1 unit increase in lnNLR = 2.51, 95% CI 1.71–3.69, p < 0.001). The association of NLR at 6 weeks and outcomes were consistent by type of ICB therapy and by line of therapy (data not shown).

### Prognostic role of a decline in NLR at 6 (±2) weeks

**Fig. 4:**
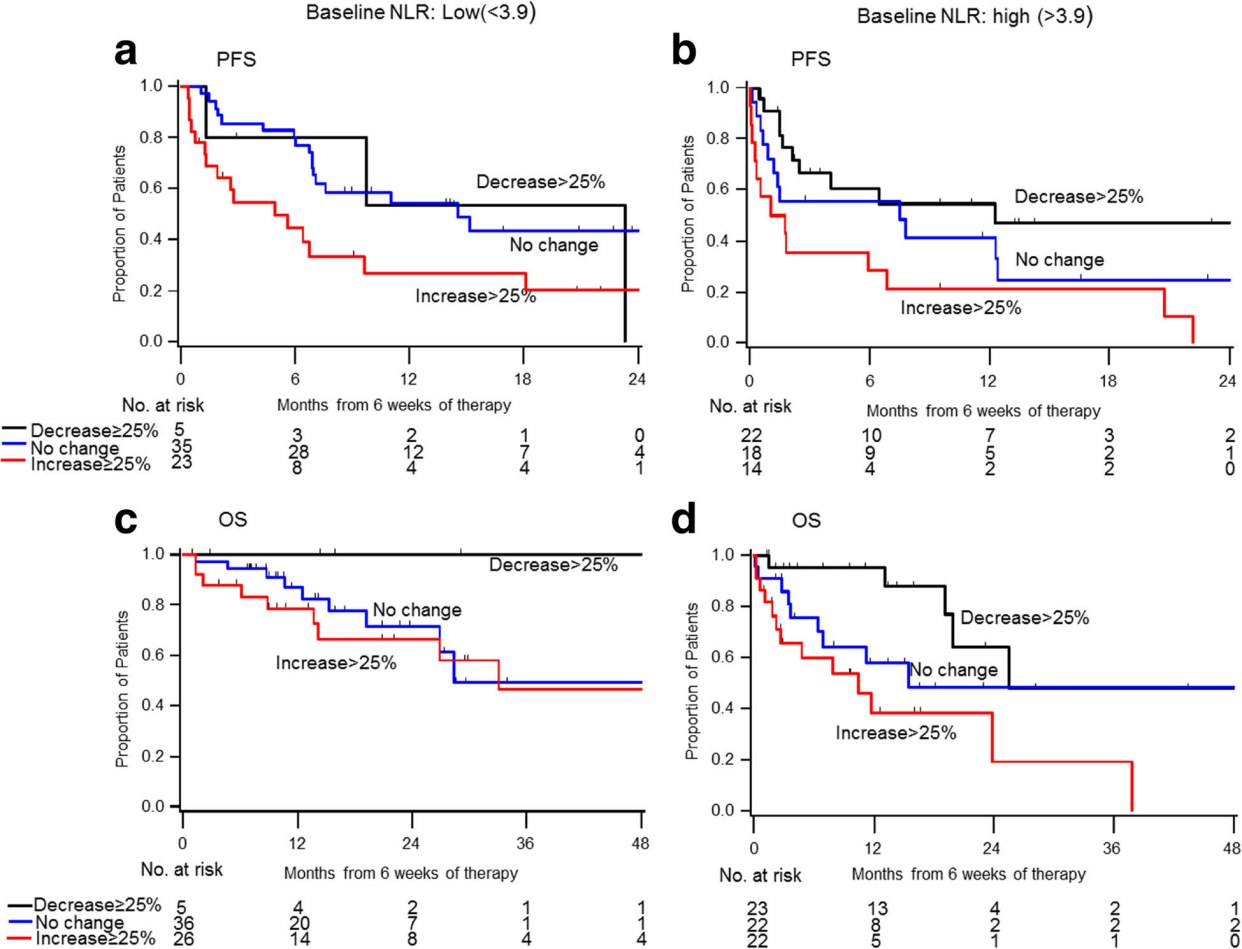
PFS and OS according to change of neutrophil-to-lymphocyte ratio (NLR) at 6 weeks, separately by baseline NLR status (Low vs High, dichotomized at the median)

Relative NLR change from baseline to 6 (±2) weeks after anti-PD-1/PD-L1 therapy was an independent prognostic factor for PFS and OS (p < 0.001 and *p* = 0.004, respectively). A decrease ≥25% was associated with an improved PFS (adjusted HR = 0.55, 95% CI 0.26–1.18) and significantly better OS (adjusted HR = 0.33, 95% CI 0.12–0.88) when compared to the no change reference group (<25% decrease to <25% increase). By contrast, an NLR increase by ≥25% was associated with significantly worse PFS (adjusted HR = 2.60, 95% CI 1.53–4.39) and OS (adjusted HR = 1.57, 95% CI 0.83–2.99). Results were consistent in subgroup analysis by baseline NLR levels (low vs high, dichotomized at the median, Fig. 4). An NLR increase by ≥25% was associated with poorer PFS and OS, regardless of baseline NLR levels.

Additionally, patients with an NLR increase ≥25% had numerically-lower ORR (Fig. 3b; OR = 0.45, 95% CI 0.18–1.16) while patients with a decrease in NLR ≥25% had numerically higher ORR rates (OR = 1.52, 95% CI 0.49–4.68), although these results were not significant (*p* = 0.112).

## Discussion

In this analysis, we show that for mRCC patients treated with contemporary PD-1/PD-L1 ICB, higher 6-week NLR was independently associated with worse outcomes in terms of reduced ORR and shorter PFS and OS. In landmark analyses, we also demonstrate that early decline (decrease ≥25%) of NLR at 6-weeks was associated with an improved PFS and significantly better OS, whereas a relative increase by ≥25% was associated with poorer PFS and OS, regardless of baseline levels. Interestingly, while the results seen at baseline (pretreatment) NLR levels were statistically significant for PFS, the numerical values for OS and ORR in this study were nearly identical to results seen in a larger NLR analysis in mRCC patients treated with VEGF-TT [15]. Taken together, our data suggests that NLR appears to be a readily-available, prognostic marker in mRCC patients treated with conventional ICB, and warrants larger, prospective validation.

Our findings are consistent with and build upon previous reports evaluating NLR in solid tumors, including RCC [12–15]. In localized RCC, higher NLR at diagnosis (> 2.7, typically pre-nephrectomy) has been shown to be associated with an increased risk of recurrence [20]. However, a review by Boissier et al. suggests that in this localized RCC setting, NLR has not been shown to be significant for overall survival based on pooled data [21]. In locally-advanced or mRCC, higher NLR (typically >3) has been shown to be an independent prognostic factor for PFS and OS [21]. However, many of these studies were performed in the context of interleukin or IFN-based regimens. In subgroup analysis of the phase III S-TRAC study, which evaluated sunitinib versus placebo in patients with high-risk locoregional RCC post nephrectomy, baseline NLR ≤ 3 was associated with improved disease-free survival (DFS) with sunitinib compared to placebo (HR 0.72, 95% CI 0.54–0.95, *p* = 0.02), whereas NLR > 3 was not (HR 1.01, 95% CI 0.58–1.77, *p* = 0.96) [22]. Templeton et al. evaluated the utility of NLR in mRCC patients primarily treated with VEGF-TT and showed that, compared with no change, increase in NLR (≥25%) at week 6 was associated with poorer OS, PFS and reduced ORR whereas an early decline (decrease ≥25%) was associated with improved outcomes [15]. Regarding treatment with contemporary ICB, studies evaluating patients with melanoma or advanced non-small-cell lung cancer (NSCLC) have shown that higher pretreatment NLR is associated with inferior OS and PFS [16–18]. However, we have performed the first analysis of the utility of NLR in mRCC patients treated in the current ICB era. Further, we similarly demonstrate the importance of changes in NLR during treatment and the prognostic relevance of measurements at week 6 independent of other factors. Given the expanding landscape and ongoing studies of PD-1/PD-L1-based therapies in mRCC [8, 23], accessible and affordable prognostic or predictive markers will continue to be a growing need.

**Fig. 5:**
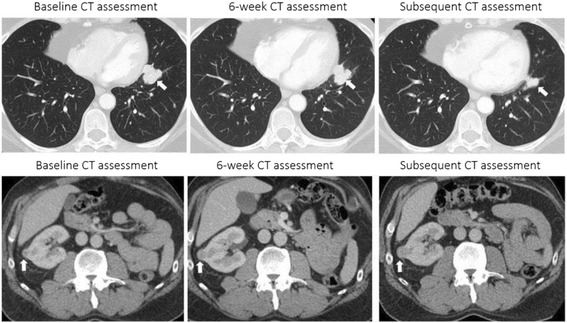
Computed tomography (CT) scans at baseline, 6-week, and subsequent assessment for two separate mRCC patients treated with PD-1/PD-L1 ICB. The first patient (upper panels) had stable disease (SD) on 6-week scan with a 34% decrease in NLR from baseline and subsequently displayed partial response (PR) on next assessment. The second patient (lower panels) had SD on 6-week scan with a 113% increase in NLR from baseline and subsequently displayed progressive disease (PD) on next assessment. Arrows (white) show change in selected area of disease burden

There are important clinical implications of our data particularly in the context of the unique and heterogenous radiographic findings in this patient population [24, 25]. While these results would benefit from prospective validation, the readouts at 6-weeks on ICB therapy are informative for both patients and physicians given that this time point typically coincides with the first set of re-staging scans after initiation of treatment. For example, if a patient presents at 6-weeks on therapy with stable or slightly progressive disease on imaging and a simultaneous decline in NLR, this may be reassuring to continue treatment assuming it is otherwise clinically suitable (Fig. 5, upper panels). Similarly, one may be more cautious regarding prognosis in a situation where a patient returns at 6-weeks with slightly progressive disease on imaging and a significant increase in NLR (Fig. 5, lower panels). Ultimately, the NLR is a helpful and available prognostic marker but should be considered in the context of other relevant clinical details when assessing the risk-benefit ratio of continuing ICB treatment at the individual patient level.

Biologically, the NLR is a marker of systemic inflammation and potentially reflects the balance of the immune system in the context of a malignancy. The neutrophil count is thought to reflect the inflammatory microenvironment that in turn has tumor-promoting activity, including cancer cell survival and proliferation, angiogenesis and metastasis, as well as subversion of adaptive immune responses [26]. Lymphocytes are effective suppressors of cancer progression and their presence, particularly in the tumor microenvironment, is thought to reflect host immunity [27]. In a study of 35 advanced RCC patients treated predominantly with IFN, Sejima et al. showed an association of Fas ligand (FasL) expression in nephrectomy tumor cells with reduced lymphocyte count and thus higher NLR [28]. They explained this association by the concept of “FasL tumor counter-attack”, whereby FasL in tumor cells mediates tumor cell immune privilege by inducing apoptosis of cytotoxic T lymphocytes in the microenvironment. On the other hand, this hypothesis has also been challenged, for example, with in vivo data [29]. Ultimately, this highlights the need for further prospective studies in patients treated with contemporary ICB, particularly with the added depth of next-generation sequencing and other informative technologies, to add more granularity to the biological underpinning of NLR in this setting.

These data should be interpreted in the context of the study design. First, this was a retrospective analysis which has the potential for selection bias and confounders. We attempted to control for this by utilizing a multivariable analysis to adjust for mRCC-specific prognostic variables that may impact analysis, including histology, line of therapy and IMDC risk factors. Our cohort included patients who were treated with PD-1 or PD-L1 ICB and there may be subtle differences between these drug pathways. For example, PD-1 inhibitors target PD-1:PD-L1 and PD-1:PD-L2, whereas PD-L1 inhibitors target PD-1:PD-L1 and PD-L1:B7.1 [30, 31]. While these slight mechanistic differences did not significantly affect our overall findings, prospective data would be informative particularly when evaluating single versus combination ICB therapy. Further, we could not control for concomitant medications that may have influenced white blood cell counts. Additionally, PD-L1 expression was not known in this retrospective analysis and may be a worthy point of future prospective study given the utility of this tissue biomarker continues to evolve in mRCC. Finally, similar to previous work investigating NLR in mRCC patients treated with VEGF-TT [15], data from untreated patients were not available in our analysis and thus it was not possible to assess the potential predictive value of NLR at baseline or on therapy.

## Conclusion

In our cohort of mRCC patients treated with PD-1/PD-L1 based immune checkpoint blockade, higher 6-week NLR was independently associated with a worse ORR and shorter PFS and OS. Early decline of NLR was associated with an improved PFS and significantly better OS, whereas a relative increase of NLR was associated with poorer PFS and OS, regardless of baseline levels. The NLR appears to be a readily-available, affordable, prognostic marker in mRCC patients treated with immune checkpoint blockade and warrants larger, prospective validation.

## Abbreviations

ccRCC: clear cell RCC
ICB: Immune checkpoint blockade
IFN-α: Interferon alfa
IMDC: International Metastatic Renal Cell Carcinoma Database Consortium (IMDC)
lnNLR: Natural log-transformed NLR
mRCC: Metastatic renal cell carcinoma
nccRCC: Non-clear cell RCC
NLR: Neutrophil-to-lymphocyte ratio
ORR: Objective response rate
OS: Overall survival
PD-1: Programmed cell death protein-1
PFS: Progression-free survival
VEGF-TT: VEGF targeted therapies
